# An example for potentially underrated causes of recessive disease in the Greater Middle East: integrative long-read genome and transcriptome sequencing pinpoint a deep-intronic homozygous *HEXB* candidate founder variant in GM2-gangliosidosis

**DOI:** 10.1186/s40246-026-00995-y

**Published:** 2026-06-10

**Authors:** Angelika Bolte, Clara Velmans, Christian Netzer, Eva Thimm, Ali Tunҫ Tuncel, Friederike Bürger, Milan Hiersche, Christian Betz, Hanno Jörn Bolz

**Affiliations:** 1 Department of Pediatrics, Pediatric Neurology, Kinderkrankenhaus Amsterdamer Strasse, Kliniken der Stadt Köln, Cologne, Germany; 2 Institute of Human Genetics, Faculty of Medicine, University Hospital of Cologne, University of Cologne, Cologne, Germany; 3 Department of General Pediatrics, Neonatology, and Pediatric Cardiology, Faculty of Medicine, University Children’s Hospital, Heinrich Heine University Düsseldorf, Düsseldorf, Germany; 4 Department of Pediatrics I, Division of Pediatric Neurology and Metabolic Medicine, Medical Faculty of Heidelberg, Heidelberg University, Heidelberg, Germany; 5 Bioscientia Bioinformatics, Bioscientia Institute for Medical Diagnostics, Ingelheim, Germany; 6 Bioscientia Human Genetics, Bioscientia Institute for Medical Diagnostics, Ingelheim, Germany

## Abstract

**Background:**

Consanguinity provides shortcuts to identify homozygous recessive mutations. However, deep-intronic variants escape standard sequencing (panel; exome/WES), and their pathogenicity cannot be inferred from genomic data. We applied WES, long-read genome and long-read-RNA-sequencing (LR-WGS, LR-RNA-Seq) in a Syrian patient with biochemically evident GM2-gangliosidosis.

**Results:**

No exonic *HEXA*, *HEXB* and *GM2A* mutations were found. LR-WGS/LR-RNA-Seq revealed a homozygous *HEXB* variant, c.771 + 985G > A, activating a 97 bp pseudo-exon.

**Conclusions:**

Integrative genome and transcriptome sequencing unlocked a deep-intronic, database-annotated *HEXB* mutation and proved causality. This illustrates the diagnostic challenges in patients from the Middle East with its prevalent consanguinity and hidden (candidate founder) mutations which are potential targets for splice-modulating therapies.

**Supplementary Information:**

The online version contains supplementary material available at 10.1186/s40246-026-00995-y.

## Introduction

GM2-gangliosidoses are a group of autosomal recessive lysosomal storage diseases. They are characterized by neurodevelopmental dysfunction and regression and result from progressive GM2-ganglioside accumulation in neuronal lysosomes due to defective catabolism of GM2-ganglioside. Three subtypes (Tay-Sachs disease, Sandhoff disease (MIM#268800), and the AB variant), caused by mutations in the genes encoding the β-hexosaminidase subunits or the GM2 activator protein–*HEXA*, *HEXB* and *GM2A*–are known. We applied WES, LR-WGS and LR-RNA-sequencing in a Syrian patient with severe neurodevelopmental regression and biochemically evident, but genetically obscure Sandhoff disease (GM2-gangliosidosis type 2), and a positive family history for the disorder. Characterization at the transcriptome level and annotations in databases qualified a deep-intronic *HEXB* variant as a pathogenic, potential founder mutation.

## Methods

Detailed methods are provided online (Supplementary Data). In brief, blood samples were obtained from the patient (for DNA and RNA analyses) and from his parents (for DNA-based segregation analysis). All investigations were conducted according to the Declaration of Helsinki, and the study was approved by the Institutional Review Board of the Ethics Committee of the University Hospital of Cologne.

Whole-exome sequencing (WES) targeted more than 20,000 human genes, including the three genes associated with GM2-gangliosidosis (*HEXA*, OMIM *606869, NM_000520.6; *HEXB*, OMIM *606873, NM_000521.4; *GM2A*, OMIM *613109, NM_000405.5) plus about 3,000 regions of particular interest (enriched with Roche KAPA HyperExome V1) and sequenced on an Illumina NovaSeq 6000.

Long-read HiFi whole-genome sequencing (LR-WGS) was performed on a PacBio Revio^®^ system, aiming at a HiFi read output of at least 80 Gb. An in-house bioinformatic pipeline which integrates long-read-specific tools (provided by PacBio (Pacific Biosciences, Menlo Park, CA) was applied. For details on variant filtering and interpretation, see Supplementary Data. Read-out of genomic data was particularly focused on GM2-gangliosidosis genes, namely *HEXA*, *HEXB* and *GM2A*, with particular attention to intronic variants. LR-WGS data were scanned for runs of homozygosity (ROH) with H3M2 [1] to estimate the degree of parental consanguinity by the ROH ratio (with values > 10% in the offspring usually indicating close parental relationship (e.g. first cousins) [2] and to define the shared outer bounds for haplotypes. For segregation analysis, both parents were genotyped for the identified *HEXB* variant.

Long-read HiFi RNA-sequencing (LR-RNA-Seq) was conducted using the Kinnex full-length RNA kit, poly(A)-mRNA without prior fragmentation was reverse-transcribed into cDNA, amplified, and concatenated to SMRTbell^®^ libraries with approximately 16 kb insert size (MAS Seq method). Sequencing was performed on a PacBio Revio^®^ system. Reads were mapped against human reference genome build GRCh38, visualized and analyzed for splicing aberrations with the Integrative Genomics Viewer (IGV) v2.19.4. [3] *HEXB* transcripts were analyzed for potential expression differences by comparison with a control cohort of 12 samples after normalization using mean expression values of selected housekeeping genes (*ATF*,* POLR2A*,* ARHGAP1*,* PRKAG1*,* ALDOA*,* ARPC4*).

cDNA PCR products amplified from reversely transcribed RNA were analyzed on a 1% agarose gel and sequenced on an ABI 3500 Genetic Analyzer (Thermo Fisher Scientific, Waltham, MA) for the patient and controls. Data were visualized using SeqPilot (JSI medical systems, Ettenheim, Germany).

Metabolic investigations included the determination of activities of β-hexosaminidase A (HEX A) and β-hexosaminidase total (HEX A + HEX B) in plasma and leukocytes by adapted methods. Activities of α-N-acetylgalactosaminidase in plasma and of β-galactosidase have been measured as reference enzymes to ensure good sample quality. Protein concentrations of leukocyte samples have been measured using an adapted method and normalized accordingly. In addition, lyso-GM2 concentration was measured in plasma using LC-MS/MS (see Supplementary Data for references with relevance for metabolic investigations).

## Results

### GM2-gangliosidosis (Sandhoff disease) in a patient from a consanguineous Syrian family with history of similar cases

The index patient was born at term (C-section) after an uneventful pregnancy with normal weight and length (no data for head circumference and APGAR). The postnatal period was normal. Unattended walking was acquired at 1 year of age. Abnormal motor development was noted at the age of 2 years. At admission to our neuropediatric unit 6 months later, it had progressed to significant muscle weakness, unsteady gait and ataxia. Ability to walk without support was lost at 30 months. At 36 months, crawling for 2–3 m and sitting were preserved, but pull to stand had become impossible. Language development had initially been normal with first adequately used words at 1 year of age. Afterwards, it was delayed with a maximum pool of 3 words. At 33 months, language use and speech comprehension were impaired.

**Fig. 1:**
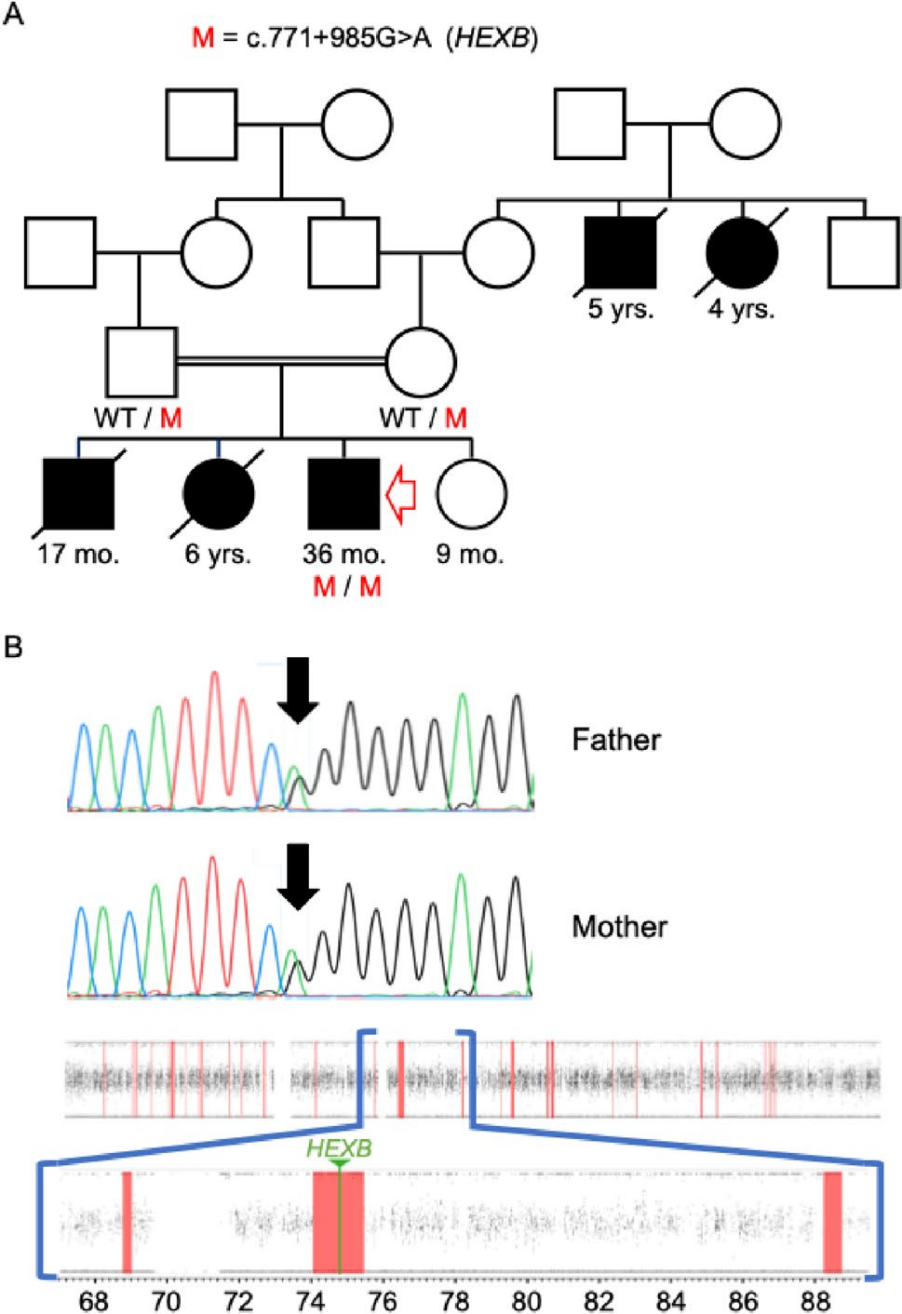
**A** Pedigree of the GM2-gangliosidosis family reported herein. Squares, male individuals; circles, female individuals; filled symbols, affected persons. Arrow, index patients whose samples were subjected to WES, LR-WGS and LR-RNA-Seq analyses. M, mutant HEXB allele (c.771 + 985G > A); WT, wild-type HEXB allele. **B** Parental segregation analyses: Electropherograms from Sanger sequencing of the respective stretch of HEXB intron 6 showing heterozygous presence of the mutant allele in both parents. **C** Plot of ROH (regions of homozygosity) on chromosome 5, zoomed in on the HEXB locus. Dots correspond to quality-filtered variants of the patient, with their genomic position (hg38, in Mb) on the x-axis and the variant allele fraction (VAF) on the y axis. Red bands mark regions called as run of homozygosity. For an overview of ROH across the genome, see Supplementary Fig. 1

The patient was born to first-cousin parents from Syria. Two older siblings had similar symptoms and died at ages of 17 months (brother) and 6 years (sister). The sister had been diagnosed with GM2-gangliosidosis through enzyme activity analysis. A younger sister (9 months of age) was healthy at the time of the last contact with the family. Biochemical and/or genetic testing was recommended to the parents, but not initiated by them since then. A maternal uncle and aunt had died at the ages of 5 and 4 years, respectively (Fig. 1A).

At 36 months of age, the child was in good general health and had a good nutritional status (weight 13,3 kg (P24), length 96 cm (P45), BMI 14,4 (P15), head circumference 50 cm (P31)). In the neuropediatric examination, it appeared alert and friendly, with responsive smile. The tongue was protruded and mouth closure incomplete. There was no active speech apart from repeated syllables, speech comprehension and playing behavior were not age-appropriate, general activity was slowed, and there was global muscular hypotonia. EEG was normal.

Biochemical analyses demonstrated a markedly elevated LysoGM2 concentration of 13,33 nmol/L in plasma (reference range 0–0.5 nmol/L). Determination of enzyme activities, as shown in Supplementary Tables 1 and Supplementary Table 2, revealed a profound reduction of β-hexosaminidase total (HEX A + HEX B) activity in plasma of 0.00 mU/ml (reference range 6.08–35.11 nmol/L) and in leukocytes of 1.38 mU/mg (reference range 6.70–26,87 mU/mg) with a concomitantly decreased β-hexosaminidase A (HEX A) activity in plasma of 0.09 mU/ml (reference range 0.45–2.12 nmol/L) and in leukocytes of 0.55 mU/mg (reference range 1.04–4.97 mU/mg). The detection of markedly elevated LysoGM2 levels, in conjunction with the combined deficiency of HEX A and HEX B activities, resulted in the diagnosis of a GM2-gangliosidosis, type Sandhoff.

### No genetic diagnosis after WES

No disease-causing variant was identified by WES with particular attention to the GM2-gangliosidosis genes *HEXA*, *HEXB* and *GM2A*.

### LR-WGS identified deep putative splice mutation in *HEXB* intron 6

**Fig. 2:**
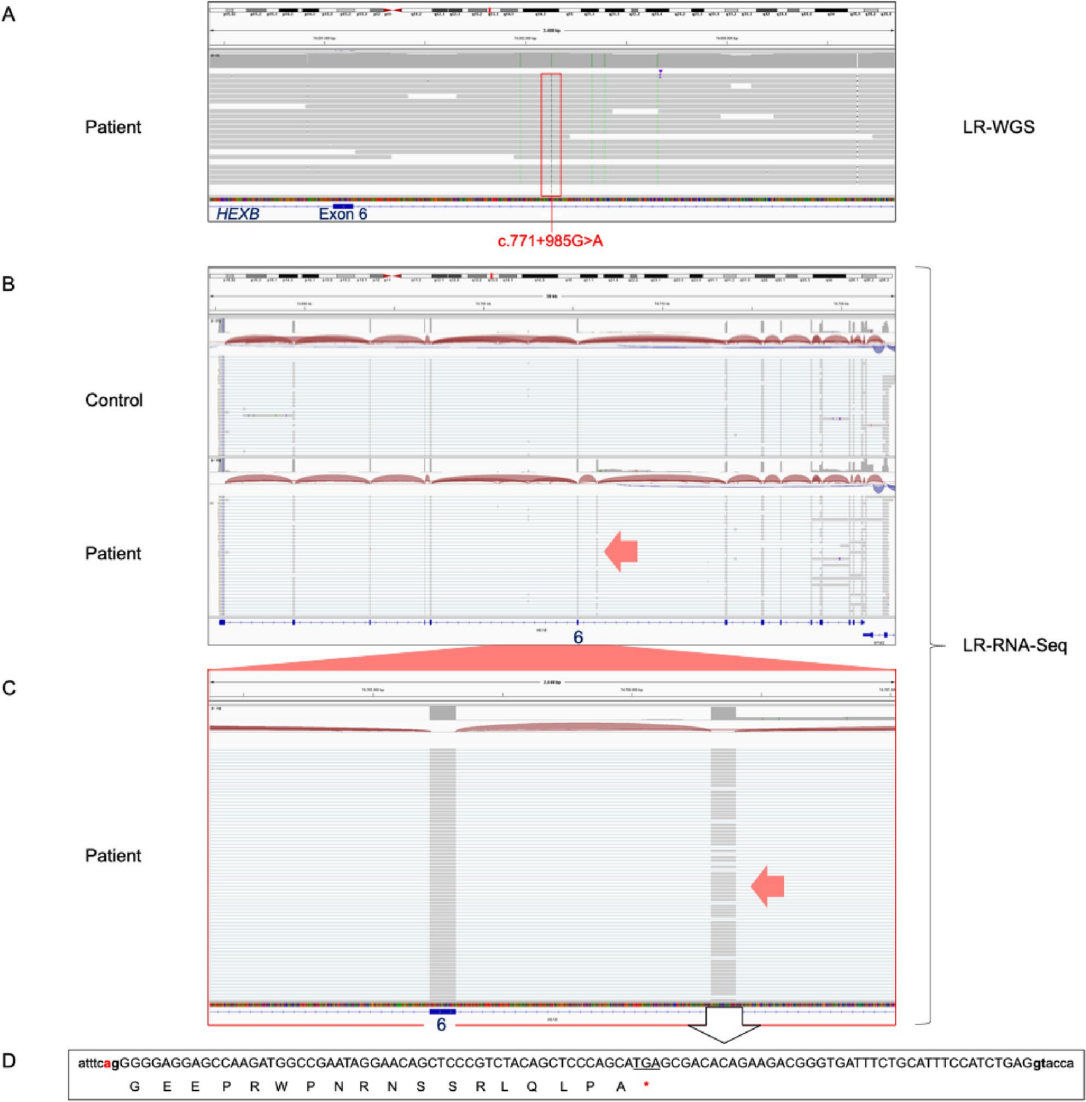
**A** LR-WGS (from Integrative Genomic Viewer, IGV) with homozygous deep-intronic HEXB variant suspected to impair splicing. All other homozygous HEXB variants (four of which are indicated in this extract) were relatively common and/or annotated as homozygous in gnomAD and not predicted to be deleterious. **B**, **C** Mapping long-read RNA-Seq reads back on the genomic HEXB sequence revealed the inclusion of 97 bp of intron 6 sequence in 94% of the reads in the patient, but not in controls. **D** The c.771 + 985G > A variant (red) introduces a novel acceptor splice site (in bold) that, with the simultaneous use of a 3’ sequence motif used as a donor splice site (in bold), resulted in the activation of a 97 bp pseudo-exon. Its inclusion into the HEXB mRNA predicts 17 unrelated residues before premature termination codon (underlined) (p.(Ser259Glufs*18))

The ROH profile derived from LR-WGS data was compatible with parental consanguinity (Fig. 1B). *HEXA* and *HEXB*, but not *GM2A*, localize in small ROH stretches. In *HEXB*, we identified a deep-intronic homozygous variant, c.771 + 985G > A (GRCh38 chr5:74706305G > A), predicted to create a novel acceptor splice site (scores: SSF 88.79, MaxEnt 5.60, NNSplice 0.41, GeneSplicer 2.84) (Fig. 2A). Both parents are heterozygous carriers of the variant (Fig. 1A). There were no rare variants in *HEXA* or *GM2A* that were predicted to be deleterious. On a genome-wide basis, there were no suspicious variants compatible with the clinical and biochemical data of our patient.

### LR-RNA-Seq reveals mis-splicing with integration of frame-shifting pseudo-exon in *HEXB* transcript

LR-RNA-Seq yielded ca. 109 Gb of transcriptome data. 102 *HEXB* full-length transcripts (NM_000521.4) were detected with an average read length exceeding 2 kb. 142 reads (with 40 reads not comprising the full-length transcript) included exon 6, 134 of which (94%) showed aberrant splicing due to c.771 + 985G > A. 8 out of 142 reads (5,6%) did not contain the pseudo-exon. The variant introduced a novel acceptor splice site which, with the simultaneous use of a 3’ sequence motif used as a donor splice site, resulted in a 97 bp pseudo-exon (Fig. 2A-D).

### c.771 + 985G > A is a loss-of-function mutation

The inclusion of 97 intron 6 nucleotides as a pseudo-exon into the *HEXB* mRNA leads to a frameshift, predicting 17 unrelated residues before termination by an in-frame premature stop codon (p.(Ser259Glufs*18); Fig. 2D). The mutation therefore results either in a heavily truncated HEXB protein (275 residues; wild-type: 556 residues) or in nonsense-mediated decay (NMD). Comparison with a normalized control cohort (*n* = 12) did not show a significant reduction of *HEXB* transcripts (NM_000521.4). However, neither truncation nor NMD would be compatible with a functional HEXB enzyme. Sanger sequencing of a cDNA PCR product comprising the region of interest confirmed the results of LR-RNA-Seq. We classified the variant as pathogenic, based on the ACMG codes PVS1 (supported by the RNA analysis results), PM2 (very low population frequency) and PS4_supportive (prevalence of the variant in further affected individuals, see below).

### c.771 + 985G > A is a potential Greater Middle East founder mutation

In addition to the annotation of our patient, there are three ClinVar submissions for the variant (July 2023: uncertain significance, zygosity not given, unknown affection status; October 2024: likely pathogenic, homozygous according to personal communication with the submitting institution, patient with Sandhoff disease; April 2025, with ACMG code PM3, implying that the variant was found heterozygous/*in trans* to a pathogenic variant, patient with Sandhoff disease). In a large-scale genome sequencing study, the variant, classified as “of uncertain significance”, was homozygous in a “4-year-old female with cherry red spot, neuroregression, seizures, and clinical features and enzyme levels suggestive of Sandhoff’s disease” [4]. The mutation is annotated as rs1210377071 in dbSNP and listed in gnomAD v.4.1.0 in heterozygous state in two individuals (one South Asian, whose WGS data are public and filed as HG03934 at the Coriell Institute, and one African/African American); no homozygous entries (152,476 alleles, MAF 1.31e-5). Alleles of dbSNP variants of the *HEXB* locus for our patient and for one of the gnomAD-listed carriers, HG03934 (female of Bengali ancestry) would at least be compatible with a shared disease-related haplotype (Supplementary Table 3); the c.771 + 985G > A could represent a Greater Middle East (GME)/Middle East-North Africa (MENA) founder mutation. However, there is currently not enough evidence to prove this hypothesis: Some of the aforementioned SNPs from the *HEXB* locus are relatively common, formal haplotype analysis could not be performed, and no information is available on the variant’s prevalence in Syria (our patient’s country of origin) or in Bangladesh – countries that are more than 5,000 km apart. Genomic ROH profile analysis revealed an ROH ratio of 6.5% (cumulative ROH size of 199.3 Mb) which is in line with the close parental consanguinity. The *HEXB* mutation resides in a very short ROH stretch of 1.3 Mb (Fig. 1B).

## Discussion

Genetic diagnostics have been revolutionized over the past 15 years through the advent of NGS, and with constant advancement of bioinformatics and sequencing technologies, accompanied by declining costs, very comprehensive testing (with WES – and increasingly even WGS – serving as first-tier tests) has become standard. Today, many patients with monogenic conditions receive diagnoses which inform about inheritance and recurrence risk, prognosis, options of personalized management and therapy.

In the family reported here, pinpointing the genetic diagnosis for this early-onset fatal condition was particularly challenging in two respects. Firstly, deep-intronic variants escape detection by approaches focusing exclusively on the coding sequence, as is the case with most NGS panels or WES. Secondly, assessing the impact of such variants’ impact on splicing requires an additional “omics layer”: transcriptome analysis. The classification of the *HEXB* variant as of “unknown significance” in two homozygous patients with a clinically compatible diagnosis (ClinVar annotation and description in reference [4]) is consistent, but illustrates the need to integrate transcriptome sequencing into routine diagnostics (together with the patients listed in ClinVar, our RNA-Seq results added sufficient evidence for a classification as “pathogenic”). As with genome sequencing, its application as long-read RNA sequencing of full-length transcripts results in “cleaner” reads which, compared to short-read approaches, align much better with reference sequences. Long RNA-Seq reads provide a realistic representation of transcript isoforms, including those from disease-related aberrant splicing (provided that the gene of interest is sufficiently expressed in the analyzed tissue, usually blood) [5,6]. From a therapy research perspective, deep-intronic splice mutations represent targets for individualized treatment approaches using splice-switching antisense oligonucleotides (ASOs) [7], which is why their exact detection and characterization at the transcriptome level is becoming increasingly important not only for diagnostic accuracy, but also for future treatments: This could also apply to the *HEXB* mutation causing GM2 gangliosidosis, a fatal disease for which studies on e.g. hematopoietic stem cell transplantation, enzyme replacement and gene therapy have not yet led to a causal treatment [8]. Furthermore, also in view of the fact that some 10% of exonic variants alter splicing [9] but may be misclassified as silent or missense variants, we consider integrative WGS and RNA-Seq as a key diagnostic approach. Due to the superiority of long-read HiFi sequencing in detecting complex (structural) variants even on a difficult sequence background (pseudogenes, repeat-enriched targets), we expect this technology to play an increasingly important role in diagnostic genomics.

Our study is of particular relevance for the Greater Middle East (GME) where (mostly recessive) pathogenic founder variants account for at least 30% of positive genetic diagnoses, causing disease often in homozygous state [10]. Such homozygosities often provide shortcuts to the genetic diagnosis, but can become pitfalls if deep-intronic. The (in at least one case homozygous) mention of the c.771 + 985G> A_*HEXB*_ variant in three additional patients with a GM2-gangliosidosis phenotype (ClinVar, reference [4]), its heterozygous annotation in two individuals in gnomAD and a putative shared *HEXB* locus haplotype in our patient and a carrier (HG03934) with public genome data could indicate a candidate GME founder allele that could be largely overlooked in patients (Supplementary Table S3). In our patient, the homozygous *HEXB* mutation resides in a short ROH stretch of just 1.3 Mb (Fig. 1C). While longer ROHs indicate recent shared ancestry (founder events or inbreeding), short ROHs can point to more distant shared ancestors and a mutation that could have originated a long time ago and has spread since then (in line with its aforementioned presence in databases and publications). Regardless of whether the variant is a true founder mutation or not, individuals with a GM2-gangliosidosis phenotype, but negative testing for this diagnosis, should be analyzed for c.771 + 985G> A_*HEXB*_, especially in case of GME origin.

Our report is an example for neglected causes of recessive disorders that may be due to variants which (using current standard diagnostic approaches) are likely to be missed or misclassified, and which – while rare elsewhere – occur in the GME to a significant extent.

## Supplementary Information

Below is the link to the electronic supplementary material.

Supplementary Material 1: Table S1. Enzyme activities in plasma. Table S2. Enzyme activities in leukocytes. Table S3. Alleles of dbSNP-annotated variants of the HEXB locus (shaded, with 5’- and 3’-prime flanking regions) for the patient reported herein and for the heterozygous carrier HG03934 (female of Bengali ancestry; gnomAD). The disease-causing variant is depicted in bold. Red: Disease-associated haplotype that is homozygous in the patient (embedded in a 1.3 Mb ROH region). Of note, this haplotype is hypothetical in HG03934 – the variants in her would at least be compatible with a haplotype shared with the patient. Figure 1. Genome-wide ROH plots from LR-WGS data. ROH stretches (red) are shown by chromosomes. Note that despite several large ROH segments (e.g. on chromosomes 3, 7, 14 and 15), the causative HEXB mutation on chromosome 5 resides in a very short ROH stretch of 1.3 Mb.

## Data Availability

The pathogenic HEXB variant reported in this paper has been submitted to ClinVar: https://www.ncbi.nlm.nih.gov/clinvar/. All other WES and WGS data are subject to controlled access to ensure the patients’ privacy.
