# Supplementary material for: An example for potentially underrated causes of recessive disease in the Greater Middle East: integrative long-read genome and transcriptome sequencing pinpoint a deep-intronic homozygous HEXB candidate founder variant in GM2-gangliosidosis
Source: Hum Genomics. 2026 Jun 10;20:96. doi: 10.1186/s40246-026-00995-y (PMC13251226; doi:10.1186/s40246-026-00995-y)
Supplement: Supplementary file 1 — Supplementary Material 1: Table S1. Enzyme activities in plasma. Table S2. Enzyme activities in leukocytes. Table S3. Alleles of dbSNP-annotated variants of the HEXB locus (shaded, with 5’- and 3’-prime flanking regions) for the patient reported herein and for the heterozygous carrier HG03934 (female of Bengali ancestry; gnomAD). The disease-causing variant is depicted in bold. Red: Disease-associated haplotype that is homozygous in the patient (embedded in a 1.3 Mb ROH region). Of note, this haplotype is hypothetical in HG03934 – the variants in her would at least be compatible with a haplotype shared with the patient. Figure 1. Genome-wide ROH plots from LR-WGS data. ROH stretches (red) are shown by chromosomes. Note that despite several large ROH segments (e.g. on chromosomes 3, 7, 14 and 15), the causative HEXB mutation on chromosome 5 resides in a very short ROH stretch of 1.3 Mb. [file 40246_2026_995_MOESM1_ESM.docx]

**SUPPLEMENTARY METHODS**

**Patients, samples**

Blood samples for DNA and RNA extraction were obtained with written informed consent. All investigations were conducted according to the Declaration of Helsinki, and the study was approved by the Institutional Review Board of the Ethics Committee of the University Hospital of Cologne. DNA and RNA were extracted by standard protocols.

**Whole-exome sequencing (WES)**

DNA of the index patient (Figure 1) was extracted from peripheral blood on a QIAsymphony instrument (Qiagen, Hilden, Germany). Genomic DNA of the patient was fragmented, and the coding exons of more than 20,000 human genes and about 3,000 regions of particular interest were enriched using Roche/KAPA sequence capture technology (Roche KAPA HyperExome V1) and sequenced on an Illumina NovaSeq 6000 system with an average coverage of 124-fold. Sequences of the three genes knowingly associated with GM2-gangliosidosis (*HEXA*, OMIM *606869, NM_000520.6; *HEXB*, OMIM *606873, NM_000521.4; *GM2A*, OMIM *613109, NM_000405.5) were retrieved from the WES data.

**Long-read HiFi whole-genome sequencing (LR-WGS)**

Short DNA fragments were removed by precipitation (SRE kit, PacBio, Menlo Park, CA), and DNA was sheared to approximately 16 kb by robot liquid handling (Hamilton NGS STAR; Hamilton, Bonaduz, Switzerland). Whole-genome library preparation and polymerase binding was done according to the protocol of the SMRTbell^®^ prep kit 3.0 (Pacific Biosciences, Menlo Park, CA). Sequencing was performed on a PacBio Revio^®^ system (1 sample per SMRT cell, 300 pM adaptive loading concentration, 24 hours movie time), aiming at a HiFi read output of at least 80 Gb.

**Genomic interpretation**

An in-house bioinformatic pipeline which integrates long-read-specific tools (HIFICNV for copy number variations; PBSV for structural variants; TRGT and TRVZ for repeat expansions) provided by PacBio (Pacific Biosciences, Menlo Park, CA) and giving additional information on phasing, methylation status and repeat expansions, was applied to process sequence data. Single nucleotide variants (SNV) were called with DeepVariant^1^ and FreeBayes (https://arxiv.org/abs/1207.3907v2). Identified variants (SNV, CNV) were matched to in-house (BiFreq) and to external databases (gnomAD,^2^ ClinVar,^3^ HGMD professional^4^). Scoring and *in silico* analyses of the variants were performed with regard to functional relevance (e.g. SIFT,^5^ PolyPhen2,^6^ MutationTaster,^7^ PROVEAN^8^), conservation (GERP,^9^ SIPHY,^10,11^ phyloP,^12^ phastCons^12^) and splicing (Alamut^13^ (integrating results from NNSplice,^14^ GeneSplicer,^15^ MaxEntScan,^16^ SpliceSiteFinder-like^17^), SpliceAI,^18^ MMSplice^19^). Basic threshold values for variant minor allele frequencies (MAF) were <0.1% for dominant inheritance and <1% for recessive diseases. Pathogenicity of rare variants was classified based on current ACMG guidelines.^20^ Read-out of genomic data was particularly focused on GM2-gangliosidosis genes, namely *HEXA*, *HEXB* and *GM2A*, with particular attention to intronic variants.

LR-WGS data were scanned for runs of homozygosity (ROH) with H3M2^21^ to estimate the degree of parental consanguinity by the ROH ratio (with values >10% in the offspring usually indicating close parental relationship (e.g. first cousins)^22^ and to define the shared outer bounds for haplotypes.

**Segregation analysis**

Both parents were genotyped for the identified *HEXB* variant. Genomic DNA from both parents was used as template for PCR with the Multiplex PCR mix (Qiagen, Hilden, Germany). Primers targeted a part of intron 6 surrounding c.771+985G>A (F: 5’-CTCAGTTAACAGTGTTCAAATTGAC-3‘; R: 5‘-CTAGTGAGATGAACCTGGTAC-3‘), yielding a PCR product of 665 bp. PCR products were analyzed on a 1% agarose gel and sequenced on an ABI 3500 Genetic Analyzer (Thermo Fisher Scientific, Waltham, MA). Subsequently, the data were visualized using SeqPilot (JSI medical systems, Ettenheim, Germany).

**Long-read HiFi RNA-sequencing (LR-RNA-Seq)**

Using the Kinnex full-length RNA kit (PacBio), poly(A)-mRNA without prior fragmentation was reverse-transcribed into cDNA, amplified, and concatenated to SMRTbell® libraries with approximately 16 kb insert size (MAS Seq method). Sequencing was performed on a PacBio Revio® system at 24 hours video time with one sample per SMRT cell. Reads were mapped against human reference genome build GRCh38, visualized and analyzed for splicing aberrations with the Integrative Genomics Viewer (IGV) v2.19.4.^23^ *HEXB* transcripts were analyzed for potential expression differences by comparison with a control cohort of 12 samples after normalization using mean expression values of selected housekeeping genes (*ATF, POLR2A, ARHGAP1, PRKAG1, ALDOA, ARPC4*).

**Sanger sequencing of cDNA PCR product**

RNA was reverse-transcribed with the RevertAid First Strand cDNA Synthesis Kit (Thermo Fisher Scientific, Waltham, MA) with primers designed to bind specifically to the poly(A) tails of the mRNA. The resulting cDNA was used as a template for PCR with the Multiplex PCR mix (Qiagen, Hilden, Germany). Primers targeted the junctions of *HEXB* exons 4/5 (forward: 5’-CAAGATTCTTATGGAACTTTCACCA-3’) and exon 7 (reverse: 5’-ATGCCCAGGGGTATCAAATTCT-3’), respectively, yielding PCR products of 342 bp (wild-type) and 439 bp (mutant). PCR products were analyzed on a 1% agarose gel and sequenced on an ABI 3500 Genetic Analyzer (Thermo Fisher Scientific, Waltham, MA). Subsequently, data were visualized using SeqPilot (JSI medical systems, Ettenheim, Germany).

**Metabolic investigations**

The activities of β-Hexosaminidase A (HEX A) and β-Hexosaminidase total (HEX A + HEX B) in plasma and leukocytes were determined by adapted methods initially decribed by Kresse et al. and Leaback et al., respectively.^24,25^ Activities of α-N-acetylgalactosaminidase in plasma and of β‑galactosidase have been measured as reference enzymes to ensure good sample quality. Protein concentrations of leukocyte samples have been measured using an adapted method described by Lowry et al.^26^ and normalized accordingly. In addition, lyso-GM2 concentration was measured in plasma using Liquid Chromatography-Mass Spectrometry (LC-MS)/MS.^27^

**References for Supplementary Methods**

1 Yun, T. *et al.* Accurate, scalable cohort variant calls using DeepVariant and GLnexus. *Bioinformatics* **36**, 5582-5589, doi:10.1093/bioinformatics/btaa1081 (2021).

2 Koch, L. Exploring human genomic diversity with gnomAD. *Nat Rev Genet* **21**, 448, doi:10.1038/s41576-020-0255-7 (2020).

3 Landrum, M. J. *et al.* ClinVar: public archive of relationships among sequence variation and human phenotype. *Nucleic Acids Res* **42**, D980-985, doi:10.1093/nar/gkt1113 (2014).

4 Stenson, P. D. *et al.* The Human Gene Mutation Database (HGMD((R))): optimizing its use in a clinical diagnostic or research setting. *Hum Genet* **139**, 1197-1207, doi:10.1007/s00439-020-02199-3 (2020).

5 Sim, N. L. *et al.* SIFT web server: predicting effects of amino acid substitutions on proteins. *Nucleic Acids Res* **40**, W452-457, doi:10.1093/nar/gks539 (2012).

6 Adzhubei, I., Jordan, D. M. & Sunyaev, S. R. Predicting functional effect of human missense mutations using PolyPhen-2. *Curr Protoc Hum Genet* **Chapter 7**, Unit7 20, doi:10.1002/0471142905.hg0720s76 (2013).

7 Schwarz, J. M., Rodelsperger, C., Schuelke, M. & Seelow, D. MutationTaster evaluates disease-causing potential of sequence alterations. *Nat Methods* **7**, 575-576, doi:10.1038/nmeth0810-575 (2010).

8 Choi, Y. & Chan, A. P. PROVEAN web server: a tool to predict the functional effect of amino acid substitutions and indels. *Bioinformatics* **31**, 2745-2747, doi:10.1093/bioinformatics/btv195 (2015).

9 Huber, C. D., Kim, B. Y. & Lohmueller, K. E. Population genetic models of GERP scores suggest pervasive turnover of constrained sites across mammalian evolution. *PLoS Genet* **16**, e1008827, doi:10.1371/journal.pgen.1008827 (2020).

10 Garber, M. *et al.* Identifying novel constrained elements by exploiting biased substitution patterns. *Bioinformatics* **25**, i54-62, doi:10.1093/bioinformatics/btp190 (2009).

11 Lindblad-Toh, K. *et al.* A high-resolution map of human evolutionary constraint using 29 mammals. *Nature* **478**, 476-482, doi:10.1038/nature10530 (2011).

12 Siepel, A. *et al.* Evolutionarily conserved elements in vertebrate, insect, worm, and yeast genomes. *Genome Res* **15**, 1034-1050, doi:10.1101/gr.3715005 (2005).

13 Houdayer, C. In silico prediction of splice-affecting nucleotide variants. *Methods Mol Biol* **760**, 269-281, doi:10.1007/978-1-61779-176-5_17 (2011).

14 Reese, M. G., Eeckman, F. H., Kulp, D. & Haussler, D. Improved splice site detection in Genie. *J Comput Biol* **4**, 311-323, doi:10.1089/cmb.1997.4.311 (1997).

15 Pertea, M., Lin, X. & Salzberg, S. L. GeneSplicer: a new computational method for splice site prediction. *Nucleic Acids Res* **29**, 1185-1190, doi:10.1093/nar/29.5.1185 (2001).

16 Shamsani, J. *et al.* A plugin for the Ensembl Variant Effect Predictor that uses MaxEntScan to predict variant spliceogenicity. *Bioinformatics* **35**, 2315-2317, doi:10.1093/bioinformatics/bty960 (2019).

17 Shapiro, M. B. & Senapathy, P. RNA splice junctions of different classes of eukaryotes: sequence statistics and functional implications in gene expression. *Nucleic Acids Res* **15**, 7155-7174, doi:10.1093/nar/15.17.7155 (1987).

18 Jaganathan, K. *et al.* Predicting Splicing from Primary Sequence with Deep Learning. *Cell* **176**, 535-548 e524, doi:10.1016/j.cell.2018.12.015 (2019).

19 Cheng, J. *et al.* MMSplice: modular modeling improves the predictions of genetic variant effects on splicing. *Genome Biol* **20**, 48, doi:10.1186/s13059-019-1653-z (2019).

20 Richards, S. *et al.* Standards and guidelines for the interpretation of sequence variants: a joint consensus recommendation of the American College of Medical Genetics and Genomics and the Association for Molecular Pathology. *Genet Med* **17**, 405-424, doi:10.1038/gim.2015.30 (2015).

21 Magi, A. *et al.* H3M2: detection of runs of homozygosity from whole-exome sequencing data. *Bioinformatics* **30**, 2852-2859, doi:10.1093/bioinformatics/btu401 (2014).

22 Gonzales, P. R. *et al.* Interpretation and reporting of large regions of homozygosity and suspected consanguinity/uniparental disomy, 2021 revision: A technical standard of the American College of Medical Genetics and Genomics (ACMG). *Genet Med* **24**, 255-261, doi:10.1016/j.gim.2021.10.004 (2022).

23 Robinson, J. T., Thorvaldsdottir, H., Wenger, A. M., Zehir, A. & Mesirov, J. P. Variant Review with the Integrative Genomics Viewer. *Cancer Res* **77**, e31-e34, doi:10.1158/0008-5472.CAN-17-0337 (2017).

24 Kresse, H., Fuchs, W., Glossl, J., Holtfrerich, D. & Gilberg, W. Liberation of N-acetylglucosamine-6-sulfate by human beta-N-acetylhexosaminidase A. *J Biol Chem* **256**, 12926-12932 (1981).

25 Leaback, D. H. & Walker, P. G. Studies on glucosaminidase. 4. The fluorimetric assay of N-acetyl-beta-glucosaminidase. *Biochem J* **78**, 151-156, doi:10.1042/bj0780151 (1961).

26 Lowry, O. H., Rosebrough, N. J., Farr, A. L. & Randall, R. J. Protein measurement with the Folin phenol reagent. *J Biol Chem* **193**, 265-275 (1951).

27 Polo, G. *et al.* Plasma and dried blood spot lysosphingolipids for the diagnosis of different sphingolipidoses: a comparative study. *Clin Chem Lab Med* **57**, 1863-1874, doi:10.1515/cclm-2018-1301 (2019).

SUPPLEMENTARY TABLES

| **Enzyme** | **Patient Result** | **Reference Range** | **Unit** |
| --- | --- | --- | --- |
| β-hexosaminidase A (HEX A) | 0.09 | 0.45 - 2.12 | mU/ml |
| β-hexosaminidase A (HEX A) (% of norm) | 10 | 49 - 229 | % |
| β-hexosaminidase total (HEX A + HEX B) | 0.00 | 6.08 - 35.11 | mU/ml |
| β-hexosaminidase total (HEX A + HEX B) (% of norm) | 0 | 48 - 275 | % |
| α-N-acetylgalactosaminidase | 0.13 | 0.06 - 0.39 | mU/ml |
| α-N-acetylgalactosaminidase (% of norm) | 85 | 39 - 257 | % |

**SUPPLEMENTARY TABLE 1**

**Enzyme activities in plasma.**

| **Enzyme** | **Patient Result** | **Reference Range** | **Unit** |
| --- | --- | --- | --- |
| β-hexosaminidase A (HEX A) | 0.55 | 1.04 - 4.97 | mU/mg |
| β-hexosaminidase A (HEX A) (% of norm) | 24 | 46 - 219 | % |
| β-hexosaminidase total (HEX A + HEX B) | 1.38 | 6.70 - 26.87 | mU/mg |
| β-hexosaminidase total (HEX A + HEX B) (% of norm) | 10 | 48 - 194 | % |
| β-galactosidase | 2.74 | 1.13 - 3.30 | mU/mg |
| β-galactosidase (% of norm) | 124 | 51 - 149 | % |

**SUPPLEMENTARY TABLE 2**

**Enzyme activities in leukocytes.**

| **Position (hg38)** | **dbSNP** | **Patient** | | **HG03934** | |
| --- | --- | --- | --- | --- | --- |
| 5:74,661,841-T-C | rs1550906 | C | C | T | C |
| 5:74,661,850-T-A | rs1006281 | A | A | T | A |
| 5:74,662,232-A-C | rs1696980 | C | C | A | C |
| 5:74,662,381-G-C | rs4704156 | C | C | G | C |
| 5:74,662,420-T-C | rs4703640 | C | C | T | C |
| 5:74,662,971-C-G | rs58047404 | G | G | C | G |
| 5:74,665,010-T-C | rs7700832 | C | C | T | C |
| 5:74,665,468-C-G | rs766318 | G | G | C | G |
| 5:74,665,784-G-C | rs820856 | G | G | C | G |
| 5:74,665,879-C-T | rs820855 | C | C | T | C |
| 5:74,666,660-C-A | rs820854 | C | C | A | C |
| 5:74,667,130-A-G | rs860245 | A | A | G | A |
| 5:74,667,327-G-A | rs820851 | G | G | A | G |
| 5:74,667,677-A-G | rs820850 | A | A | G | A |
| 5:74,668,293-T-C | rs11958029 | C | C | T | C |
| 5:74,668,294-G-A | rs11960249 | A | A | G | A |
| 5:74,668,835-A-G | rs820848 | A | A | G | A |
| 5:74,669,546-C-G | rs17646591 | C | C | G | C |
| 5:74,670,862-C-T | rs1665892 | C | C | T | C |
| 5:74,698,270-T-C | rs577732102 | C | C | T | C |
| 5:74,698,633-G-A | rs545271302 | G | G | A | G |
| **5:74,706,305-G-A** | **rs1210377071** | **A** | **A** | **G** | **A** |
| 5:74,736,364-G-A | rs529182105 | A | A | G | A |
| 5:74,742,814-G-A | rs547654983 | G | G | A | G |
| 5:74,744,247-T-A | rs528516871 | A | A | T | A |
| 5:74,747,325-C-G | rs570597381 | G | G | C | G |
| 5:74,770,561-A-G | rs1164694 | A | A | G | A |
| 5:74,779,604-C-G | rs1164692 | C | C | G | C |
| 5:74,797,617-T-C | rs567082750 | C | C | T | C |
| 5:74,818,805-A-C | rs36077153 | C | C | A | C |
| 5:74,823,901-C-G | rs1146146 | C | C | G | C |
| 5:74,828,054-T-C | rs1164625 | T | T | C | T |
| 5:74,832,087-T-G | rs1748341894 | T | T | G | T |
| 5:74,836,892-G-A | rs1164626 | G | G | A | G |
| 5:74,878,694-A-G | rs559028781 | G | G | A | G |
| 5:74,947,027-A-G | rs3934476 | G | G | A | G |
| 5:74,947,059-T-G | rs4075707 | G | G | T | G |
| 5:74,947,295-A-C | rs7356745 | C | C | A | C |

**SUPPLEMENTARY TABLE 3**

**Alleles of dbSNP-annotated variants of the *HEXB* locus (shaded, with 5’- and 3’-prime flanking regions) for the patient reported herein and for the heterozygous carrier HG03934 (female of Bengali ancestry; gnomAD).** The disease-causing variant is depicted in bold. Red: Disease-associated haplotype that is homozygous in the patient (embedded in a 1.3 Mb ROH region). Of note, this haplotype is hypothetical in HG03934 – the variants in her would at least be compatible with a haplotype shared with the patient.

SUPPLEMENTARY FIGURES

**SUPPLEMENTARY FIGURE 1**

Genome-wide ROH plots from LR-WGS data. ROH stretches (red) are shown by chromosomes. Note that despite several large ROH segments (e.g. on chromosomes 3, 7, 14 and 15), the causative *HEXB* mutation on chromosome 5 resides in a very short ROH stretch of 1.3 Mb.
